# Identification of astrocytoma associated genes including cell surface markers

**DOI:** 10.1186/1471-2407-4-39

**Published:** 2004-07-21

**Authors:** Kathy Boon, Jennifer B Edwards, Charles G Eberhart, Gregory J Riggins

**Affiliations:** 1 Department of Neurosurgery, Mason F. Lord Bldg., Center Tower, 5^th ^Floor, 5200 Eastern Avenue, Baltimore MD 21224, USA; 2 Department of Pathology, Johns Hopkins University School of Medicine, Baltimore, MD 21205, USA

**Keywords:** SAGE, astrocytoma, gene expression, bioinformatics and therapeutic targets.

## Abstract

**Background:**

Despite intense effort the treatment options for the invasive astrocytic tumors are still limited to surgery and radiation therapy, with chemotherapy showing little or no increase in survival. The generation of Serial Analysis of Gene Expression (SAGE) profiles is expected to aid in the identification of astrocytoma-associated genes and highly expressed cell surface genes as molecular therapeutic targets. SAGE tag counts can be easily added to public expression databases and quickly disseminated to research efforts worldwide.

**Methods:**

We generated and analyzed the SAGE transcription profiles of 25 primary grade II, III and IV astrocytomas [1]. These profiles were produced as part of the Cancer Genome Anatomy Project's *SAGE Genie *[2], and were used in an *in silico *search for candidate therapeutic targets by comparing astrocytoma to normal brain transcription. Real-time PCR and immunohistochemistry were used for the validation of selected candidate target genes in 2 independent sets of primary tumors.

**Results:**

A restricted set of tumor-associated genes was identified for each grade that included genes not previously associated with astrocytomas (e.g. VCAM1, SMOC1, and thymidylate synthetase), with a high percentage of cell surface genes. Two genes with available antibodies, Aquaporin 1 and Topoisomerase 2A, showed protein expression consistent with transcript level predictions.

**Conclusions:**

This survey of transcription in malignant and normal brain tissues reveals a small subset of human genes that are activated in malignant astrocytomas. In addition to providing insights into pathway biology, we have revealed and quantified expression for a significant portion of cell surface and extra-cellular astrocytoma genes.

## Background

Astrocytomas are the most frequent malignant primary brain tumors in adults. Clinically, this group of tumors can be divided into four World Health Organization (WHO) grades. Pilocytic astrocytomas (WHO grade I) are generally slow growing and non-infiltrative pediatric tumors, which are rarely fatal. For the infiltrating astrocytomas, survival decreases with increasing grade. Grade II astrocytomas patients survive an average of over 5 years, but survival drops to 3 years for anaplastic astrocytomas (grade III). Grade IV astrocytomas (glioblastoma multiforme or GBM) account for about half of all astrocytic tumors, with a median survival of less than a year. Effective treatment options for the invasive grade II to IV tumors are still limited to surgery and radiation therapy, with most chemotherapy regimens showing little or no increase in survival.

Several recent gene expression profiling studies of human astrocytomas have been able to distinguish between various grades of astrocytomas and between astrocytomas and other human glial tumors, and to identify new molecular classes within histological grade [3-7]. This enhanced molecular classification based on expression patterns of genes and pathways holds promise for better diagnostic and prognostic tools. Candidate glioblastoma associated genes have also been identified using expression profiling [8-11]. While these studies in brain cancers have produced leads for potential therapeutic targets, a systematic and comprehensive evaluation of gene expression in malignant astrocytomas is not readily and freely available for the scientific community.

In this study, we sought to create a public and comprehensive gene expression resource for astrocytomas, with the primary intention of aiding searches for new therapeutic targets in malignant astrocytomas. For this purpose we produced and analyzed in detail 25 gene expression profiles of primary astrocytic tumors (grade II, III and IV) using Serial Analysis of Gene Expression (SAGE) [12]. Complete expression profiles are posted for the scientific community at the CGAP SAGE Genie website [2]. The utility of the resource was validated by extensive comparisons of tumor with normal tissue. SAGE profiles on normal brain and other tissues created by the Cancer Genome Anatomy Project [8] were used to subtract out the genes normally expressed in adult brain, leaving a small and specific set of astrocytoma associated genes for each class, and revealing cell surface or extra-cellular matrix related genes highly expressed in the tumors when compared to their expression in normal tissue. A subset of the tumor-associated genes was validated in an independent set of tumors at both the transcript and protein level. In summary we have identified several novel tumor-associated markers in astrocytic tumors as well as various cell surface markers highly expressed in the most aggressive tumor types.

## Methods

### Tissue and RNA

Astrocytic tumor samples from 21 adults and 4 children were obtained from the Duke Brain Tumor Bank. All samples were classified based on histology according to the World Heath Organization grading criteria. Pediatric normal cortex (15 months) was a gift of Dr. Rachel Myerowitz and normal pediatric cerebellum was from the Maryland Brain Bank. Normal adult cortex and cerebellum were rapid autopsy samples obtained from the Duke Alzheimer's Brain Bank. Total RNA from normal substantia nigra was obtained from Clontech (Palo Alto, CA). PolyA+ RNA from normal adult leukocytes was obtained from Stratagene (La Jolla, CA), as noted for each library's information at the SAGE Genie Website [2]. RNA integrity was confirmed by gel electrophoresis prior to SAGE library construction.

### SAGE libraries and informatics

SAGE libraries from 25 selected astrocytomas: 8 grade II astrocytomas, 10 grade III anaplastic astrocytomas and 7 grade IV glioblastomas (primary GBM) were constructed using *Nla *III as the anchoring enzyme and *BsmF *I as the tagging enzyme using a micro-SAGE protocol. The SAGE library clones were partially arrayed at Lawrence Livermore National Laboratories and inserts were purified and sequenced at the BC Cancer Agency Genome Sciences Centre or arrayed and sequenced at Agencourt Bioscience Corporation.

The SAGE 2000 software version 4.12 (available at ) was used to extract SAGE tags from the original sequence files, remove duplicate ditags, remove linker sequences, remove one base pair variations of linker sequences and tabulate the occurrence of each tag. Tag sequences, tag counts and gene associations were stored in a Microsoft Access relational database for subsequent selection of tags with a particular profile. A total of 2,605,122 tags were obtained with an average of 102,988 tags per library. Normal neural tissue tags included a total of 443,560 tags from normal brain [8,13]. These SAGE normal brain libraries have the following unique identifiers: cortex_B_BB542; cortex_B_pool6; thalamus_B_1; cerebellum_B_1; cerebellum_B_BB542; peds_cortex_B_H1571 and substantia_nigra_B_1. Tags totaling 48,039 were also included from normal leukocytes (SAGE_Leukocytes_normal_B_1). Detailed library information and tag counts for each tissue are located at CGAP's SAGE Genie [2]. Tag counts were normalized to 100,000 tags per library.

### Real-time PCR

Total RNA extraction, cDNA synthesis and quantitative PCR were performed as previously described [1]. Gene expression levels were normalized to 3 genes; GAPDH, ribosomal protein RPS27 and low molecular mass ubiquinone-binding protein (QP_C). Both RPS27 and QP-C showed a relatively even expression level across the libraries as assed by SAGE analysis. Relative expression levels were calculated in comparison to the levels in nine normal neural tissues, including normal brain (3x), cerebellum (2x), thalamus, gray matter, caudate nucleus and pediatric cortex according to Saha *et al*., 2001 [14]. A list of the PCR primers used for each gene is available upon request.

### Immunohistochemistry

Formalin-fixed 5-μm paraffin-embedded sections were stained with various antibodies using the biotin/streptavidin RTU Vectastain Universal Quick kit (Vector laboratories, Burlingame, CA) as previously described [1]. Shortly, sections were deparaffinized in HemoD and re-hydrated through descending alcohols. Endogenous peroxidase was quenched by incubating the slides in methanol/5% H_2_O_2 _at room temperature for 10 min. Non-enzymatic antigen retrieval was performed using the Antigen retrieval solution AR or Citra (Biogenex, San Ramon, CA) in combination with microwave treatment. Sections were then blocked in PBS/0.5% Triton X-100 containing 2.5% normal horse serum for at least 30 min at room temperature and incubated with primary antibody overnight at 4°C. The primary antibodies used were mouse monoclonal anti-aquaporin1, at a 1:60 dilution (Abcam Limited, Cambridgeshire, United Kingdom) and mouse monoclonal anti-Topoisomerase II Alpha used at a 1:30 dilution (Novocatra Laboratories, New Castle, United Kingdom). Sections were developed with a DAB (Sigma, St. Louis, MO) substrate, counterstained with hematoxylin and mounted with Cytoseal-60.

### Tissue micro arrays

The tissue micro array used in these studies contained cores from 20 glioblastomas, 20 anaplastic astrocytomas, 20 infiltrating astrocytomas and 20 oligodendroglial lesions and was prepared according to methods described by Kononen and Kallioniemi [15]. Microscopic examination of the array confirmed that the appearance of the tumor tissue cores corresponded to that in the donor blocks. A neuropathologist (CGE) selected the tumor areas sampled in each case and examined the resulting arrays to ensure they accurately represented the donor cases.

## Results and Discussion

### SAGE gene expression profiles, selection and confirmation of tumor-associated genes

This report describes the comprehensive generation of expression profiles of three astrocytic tumor grades based on Serial Analysis of Gene Expression (SAGE). The main goals were to identify genes not expressed in normal brain tissue and genes highly expressed in the more aggressive astrocytomas encoding cell surface or extra-cellular matrix related proteins that could be of potential therapeutic interest. We generated SAGE profiles on 8 infiltrating astrocytomas, 10 anaplastic astrocytomas and 7 glioblastoma samples. Combined with two glioblastoma profiles [8], previously deposited on SAGE Genie, this study analyzed 2,734,106 astrocytoma SAGE tags. On average we could distinguish over 27,000 unique tags in each tumor grade after excluding those with single counts (Table 1). Complete expression profiles and library information are posted for the scientific community at the Cancer Genome Anatomy Project (CGAP) SAGE Genie website , where the libraries can be downloaded or viewed online using SAGE Genie tools [2].

In order to identify tumor-associated genes, we sought highly expressed transcripts in each grade of astrocytoma that were not expressed in 7 normal brain tissues. This also helped control for contaminating normal cells within the tumor sample. Tags expressed in a normal leukocyte SAGE library were also included in the analysis so they could be subtracted to reduce the chances of identifying transcripts from white blood cells that frequently infiltrate these tumors. Initially we selected for tags with an average expression of at least 3 per 100,000 tags. Subsequently, we selected for tags expressed at less than 2 counts per 100,000 in each of the 8 normal libraries (7 normal neural tissues & one leukocyte), reducing the number of tags to less than 100 per tumor type (Table 1). We further narrowed down the list of candidates by including only those tag sequences that could be matched with a full-length cDNA sequence. From these lists of genes we selected respectively 8, 16 and 10 genes for real-time PCR analysis in an independent set of 14 to 17 grade II, grade III and grade IV primary tumors. Only 6 genes could not be confirmed by real-time PCR analysis, 3 of 8 (grade II selection) and 3 of 16 (grade III selection). Another 8 genes were confirmed in only 20 to 25 % of the tumors. Table 2 lists those genes with a 5-fold or more over-expression by real time PCR in at least 30% of the tumor samples from the corresponding grade, when compared to an average of the normal neural tissue expression. The results show that the *in silico *selection using SAGE profiles revealed tumor-associated genes that can be found in a different set of primary tumors implying a possible role for these genes in tumor development and increasing their value as putative therapeutic targets.

**Figure 1:**
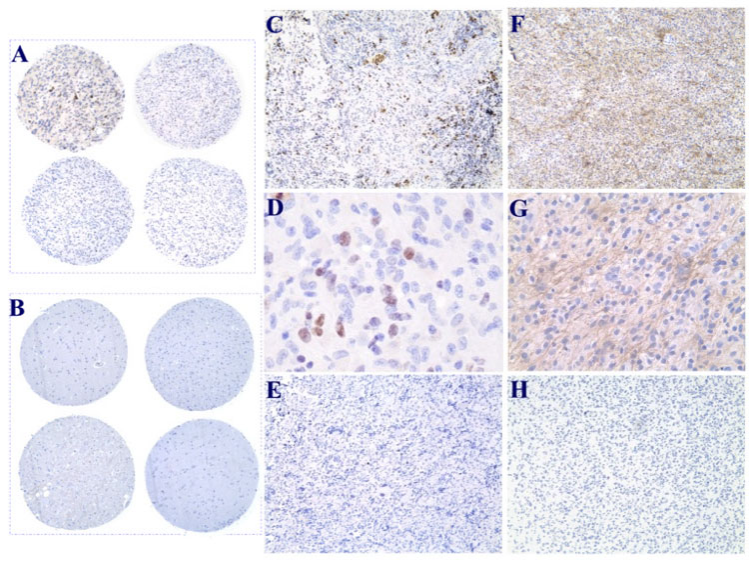
**Representative immunostaining of paraffin sections with antibodies against Topoisomerase 2A and Aquaporin 1. **(**A**) Upper panel shows two TOP2A positive cores from GBMs; lower panel shows two negative cores on the same tissue micro-array. (**B**) Upper panel from left to right: cortex and white matter; lower panel from left to right: spinal cord and hypocampus. All stained with anti-TOP2A antibodies. (**C **– **D**) Paraffin section of GBM showing nuclear staining of TOP2A; **D **shows a higher magnification and **E **is the corresponding negative control with mouse IgG1 and no primary antibodies. (**F **– **G**) Paraffin section of GBM showing cytoplasmatic staining with anti-aquaporin 1 antibodies; **G **shows a higher magnification and **H **is the negative control.

The limited availability of high quality antibodies for the identified tumor-associated genes (Table 2) narrowed our study at the protein level to those genes that had previously been implicated in astrocytic tumors. Monoclonal antibodies for *TOP2A *and *AQ1 *were used to analyze the expression at the protein level in individual glioblastoma sections and in a tissue micro-array. Strong nuclear staining in 5 of 8 individual glioblastomas tested was found for TOP2A (Figure 1A,1C and 1D). The GBMs showed intensely staining cells with the percentage of positive cells varying between 3 and 10%. Similar results were obtained when staining a tissue micro-array containing a different set of 20 GBMs. In six cases we found 5 to 10% of the cells expressing TOP2A. The percentage of strong positive cells among the 20 anaplastic astrocytomas present on the tissue micro array was lower and estimated to be 2 – 3% in 10 cases. Normal neural brain tissues like cortex, white matter, spinal cord and hippocampus where negative for TOP2A (Figure 1B). Cytoplasmatic staining was observed when anti-aquaporin 1 monoclonal antibodies were used. Three of 5 individual GBM sections where positive, with examples shown in Figures 1F and 1G. These results emphasize that the high transcript levels of TOP2A and Aquaporin 1 correlate well with a high protein level in a third independent set of tumors, implying that this might also be the case for the other identified tumor-associated genes as listed in Table 2 and Table 3.

### Potential therapeutic targets in astrocytic tumors

Encouraged by the confirmation of our initial *in silico *analysis for tumor-associated genes we formulated a slightly simplified approach to find cell surface, extra-cellular matrix and cell adhesion related genes. We selected for transcript tags with at least a ten fold over-expression when compared to the average expression level in normal neural brain tissues. Next we applied a filter that would include only those tags with an average expression of at least 5 or 10 counts per 100,000 tags in 30% or more of the tumors, respectively for anaplastic astrocytomas and glioblastomas. The generated lists of transcript tags were mapped to the corresponding gene using SAGE Genie, where after the gene ontology information (if available) was used as a final filter to identify membrane, cell surface and cell adhesion related genes (Table 3). Interestingly, almost 50% of the genes identified as highly expressed in both tumor types have not previously been implicated in astrocytomas and are potential new therapeutic targets.

Intracellular proteins that contribute to the fusion of the vesicles with the plasma membrane during exocytosis include synaptosomal protein and vesicle-associated membrane proteins (VAMP). Both anaplastic astrocytomas and glioblastomas show high expression of VAPB and anaplastic astrocytomas express caveolin1 (Table 3). It has been shown that caveolae require intact VAMP for targeted transport in endothelial cells. Caveolae and associated proteins might be targeted in cancer as recently suggested [16].

One of the other genes expressed in common between the three astrocytomas is chitinase 3-like 2 (*CHI3L2*) or *YKL-39 *(Table 2). *CHI3L2 *is a chondrocyte growth related gene and is an antigen found in rheumatoid arthritis [17] and osteoarthritis and a possible immunotherapy target. Another commonly over-expressed gene is *Neuromedin B*. This neuropeptide has been implicated as an autocrine growth factor in lung cancer cells [18] that binds to a G protein-coupled receptor on the cell surface and might have a similar role in astrocytic tumors. It is tempting to speculate that a specific neuropeptide antagonist or neutralizing antibodies might reduce astrocytoma growth. Neuromedin B had previously been described as a GBM marker [9], and was included along with *ABCC3 *in the real-time PCR analysis as a positive control.

Another relatively unknown gene is the recently characterized *SMOC-1 *[19], which was identified as a grade II and III tumor-associated gene (Table 2). This gene is related to SPARC/osteonectin, which was reported to participate in angiogenesis and tumor formation of human melanomas. Another extra cellular gene, Matrix Gla protein [20] had increased expression levels in higher-grade astrocytomas. MGP helps regulate the calcification of the extra cellular matrix [20].

Aquaporin 1 is an integral membrane protein important in the regulation of water transport in various epithelial and endothelial cell types [21]. The over-expression of *AQP1 *in human brain tumors was described in a limited array study of 4 Glioblastomas [11] and it has been suggested that the protein might play a role in brain tumor edema in a similar way as the closely related aquaporin-4 [22]. Although the specific role of AQP1 in brain tumors is still unknown, our demonstration that AQP1 is consistently expressed in GBM may prompt other studies.

Thymidilate synthetase and Topoisomarese 2A were over-expressed in glioblastoma as well as in our previous study of medulloblastoma [1]. Considering the role of Top2A as a molecular target of various anticancer drugs, and its identification as a survival marker in astrocytomas [23], its over-expression at the protein level in multiple brain tumors and the development of TOP2A inhibitors [24] makes the molecular targeting of TOP2A worthy of further investigation.

In summary we have identified a number of new tumor-associated genes for three different grades of astrocytic tumors, and helped re-confirm in a larger set of samples several previously known astrocytoma genes. Despite the high heterogeneity among gliomas, a small set of genes is consistently observed at high levels in more than a third of each grade of astrocytoma studied. Many other cell surface, extra-cellular matrix or cell adhesion genes have been identify as potential targets for cancer therapy in astrocytic tumors. Although the therapeutic value of these markers is speculative at this point, by integrating this data onto the commonly used gene expression resource, *SAGE Genie*, this data can be used as a standard to determine gene expression in astrocytomas. Further evaluation by *in vitro *and *in vivo *studies will be necessary to establish the role of these over-expressed genes in brain tumor development and progression.

## Authors' contributions

KB performed computational analyses, generated experimental data, participated in the design of the study and drafted the manuscript. JBE generated experimental data. CBE and GJR participated in the study design and manuscript editing. All authors read and approved the final manuscript.

## Pre-publication history

The pre-publication history for this paper can be accessed here:



## Figures and Tables

**Table 1:** Summary of number of tag sequences.

**Group**	**Total tags**	**Unique tags^a^**	**Expressed tags per library^b^**	**Expressed in tumors and not in neural normals^c^**
Grade II	869,992	22,574	3,999	41
Grade III	1,026,620	26,445	3,866	55
Grade IV	798,451	33,541	3,518	76

**^a^**Unique tags with a tag count of 2 and higher. **^b^**Amount of tags with an expression level of 3 or more per 100,000 tags. **^c^**Amount of tags expressed in at least 20% of the tumors and with an expression level of 2 or less in the normal neural tissues and leukocytes.

**Table 2:** Astrocytoma associated genes validated by real-time PCR.

**Tag sequence**	**Symbol**	**Gene/Ontology**	**Mean tumor^a^**	**Mean normal^b^**	**% SAGE^c^**	**% RT-PCR^d^**
		***Low grade astrocytomas (grade II)***				
TGGGATTCCC	*CHI3L2*	Chitinase 3-like 2 / cartilage biogenesis, extra-cellular	10.8	0	25	57
ATCTGCTCGG	*NMB*	Neuromedin B / signal transduction, neuropeptide	9.1	0.1	38	57
GTCAGAACTT		cDNA FLJ22528 / unknown	5.8	0.3	38	43
GTACGGAGAT	*VCAM1*	Vascular cell adhesion molecule 1/ cell adhesion	4.4	0.2	50	29
		***Anaplastic astrocytomas (grade III)***				
TGGGATTCCC	*CHI3L2*	Chitinase 3-like 2 / cartilage biogenesis, extra-cellular	12.3	0	30	63
GTGCGGAGGA	*SAA1*	Serum amyloid A1/ inflammatory response, lipid metabolism, extra-cellular	11.4	0	20	50
TTTGCACTTT	*SMOC1*	Secreted modular calcium-binding 1/ calcium ion binding	6.0	0	50	50
CCATTGTGGA	*ADORA3*	Adenosine A3 receptor / signal transduction, G-protein coupled receptor	4.1	0.1	60	50
ATCTGCTCGG	*NMB*	Neuromedin B/ signal transduction, neuropeptide	9.1	0.1	40	81
CGCGGCGGCG	*CRF*	C1q-related factor / function unknown	7.8	0.1	70	56
CCCAGTAAGA	*CSRP2*	Cysteine and glycine-rich protein 2 / cell differentiation/growth, LIM domain protein	9.4	0.2	80	44
GTACGGAGAT	*VCAM1*	Vascular cell adhesion molecule 1/ cell adhesion	3.0	0.2	20	69
		***Glioblastomas (grade IV)***				
ACTGTCGCCA	*AQP1*	aquaporin 1 / water transport, ion channel	5.8	0	22	53
ATGTAGAGTG	*TYMS*	thymidylate synthetase / pyrimidine metabolism	7.1	0	67	71
CTCAGCAATG	*TOP2A*	topoisomerase (DNA) II alpha (170 kD) / DNA metabolism, isomerase	4.2	0	44	88
GTCAACAGTA	*ABCC3*	ATP-binding cassette, subfamily C, member 3/ multidrug resistance, transport protein	12.3	0	89	94
GTGCGGAGGA	*SAA1*	serum amyloid A1/ inflammatory response, lipid metabolism, extra-cellular	14.3	0	44	53
TGGGATTCCC	*CHI3L2*	chitinase 3-like 2 / cartilage biogenesis, extra-cellular	31.3	0	67	100
ATCTGCTCGG	*NMB*	Neuromedin B / signal transduction, neuropeptide	4.6	1	44	53
GTTTATGGAT	*MGP*	Matrix Gla protein /cartilage condensation, ossification, extra-cellular	7	2	56	35

All counts are normalized per 100,000 tags. **^a^**Average tag count per individual tumor SAGE library. **^b^**Average tag count per individual normal neural tissue SAGE library. **^c^**Percentage of tumor SAGE libraries with an expression of at least 5 or more counts. **^d^**Percentage of tumor samples in an independent panel of tumors with at least a 5-fold of more over-expression when compared to neural normal tissue samples in real-time PCR analysis.

**Table 3:** Selected highly expressed genes in grade III and IV astrocytomas.

**Tag Sequence**	**Tumor^a^**	**Induction^b^**	**Gene**	**Gene Ontology**
			***Anaplastic astrocytomas (Grade III)***	
AAGCCGAAGA	10	40	GFAP glial fibrillary acidic protein	Major intermediate filament protein of mature astrocytes
CTGGGGAGTG	8.1	22	VAPB VAMP associated protein B and C	Type IV membrane protein in plasma and intracellular vesicle membranes
GCAACAGCAA	80.9	19	SEC61G Sec61 gamma subunit	Endoplasmatic reticulum membrane protein, translocation
TTAATCTGAG	6.9	18	ARHGEF6	Rac/Cdc42 guanine nucleotide exchange factor (GEF) 6, intra-cellular
ATCTTGTTAC	11	15	FN1 fibronectin 1	Extra-cellular matrix, cell adhesion, cell migration
GGAACAAACA	11.9	14	CD24	Plasma membrane, small cell lung carcinoma cluster 4 antigen
AGGGAGGGGC	32.9	14	GPX3 glutathione peroxidase 3	Extra-cellular, response to oxidative stress, glutathione metabolism
GTATGGGCCC	26.8	13	CHI3L1 chitinase 3-like 1	Cartilage glycoprotein
ACGAGGGGTG	71.4	12	BCAN, chondroitin sulfate proteoglycan	Cell adhesion
CACTTGAAAA	6.9	11	CALU calumenin	Endoplasmatic reticulum, calcium ion binding
GATAACTACA	6.7	11	IGFBP7	Extra-cellular, insulin-like growth factor binding protein 7
TCCTGTAAAG	6.8	11	CAV1 caveolin 1, 22 kDa	Plasma membrane, caveolae protein, integrin-mediated celladhesion, vesicular trafficking
GTCTTAAAGT	41.8	10	SOD2	Superoxide dismutase, mitochondrial, erythropoietin-mediated neuro protection through NF-kB
			***Glioblastomas (grade IV)***	
GTATGGGCCC	144.2	72	CHI3L1 chitinase 3-like 1	Cartilage glycoprotein
GACCACCTTT	17.3	69	MFAP2 microfibrillar-associated protein 2	Major antigen of elastin-associated microfibrils
ACCAAAAACC	115.8	49	COL1A1 collagen, type I, alpha 1	Extra-cellular matrix
AGTGGTGGCT	14.8	39	FMOD fibromodulin	Extra-cellular matrix, TGF beta receptor complex assembly
CTGGGGAGTG	13.2	35	VAPB VAMP associated protein B and C	Type IV membrane protein in plasma and intracellular vesicle membranes
ACATTCTTTT	38.2	34	GPNMB glycoprotein nmb	Transmembrane glycoprotein
ATCTTGTTAC	21.1	28	FN1 fibronectin 1	Extra-cellular matrix, cell adhesion, cell migration
GCAACAGCAA	97.9	23	SEC61G Sec61 gamma subunit	Endoplasmatic reticulum membrane protein, translocation
TCACCAAAAA	19.3	19	STAB1 stabilin 1	Cell adhesion, calcium ion binding
CACTTGAAAA	11.6	19	CALU calumenin	Endoplasmatic reticulum, calcium ion binding
TTTTCAAATA	15.4	15	T1A-2 lung type-I	Type I integral membrane glycoprotein
GTCTTTCTTG	24.4	14	RAB13	Cell adhesion, member RAS oncogene family
GGAAATGTCA	12.6	13	MMP2 matrix metalloproteinase	Extra-cellular matrix, calcium ion binding
GTACTAGTGT	23.8	12	CCL2 chemokine (C-C motif) ligand 2	Extra-cellular matrix, cell adhesion, signal transduction
AAGCTGTATA	16.7	12	TNC tenascin C (hexabrachion)	Extra-cellular matrix, cell adhesion
GTGCTAAGCG	10.8	11	COL6A2 collagen, type VI, alpha 2	Extra-cellular matrix
CATATCATTA	151.6	11	IGFBP7	Extra-cellular, insulin-like growth factor binding protein 7
AACACAGCCT	24.9	11	C4A complement component 4A	Extra-cellular matrix, complement pathway

Highly expressed genes were selected with at least a ten fold over-expression when compared to normal neural tissue and a minimal expression level of at least 5 or 10 counts per 100,000 tags (in respectively grade III and IV tumors) in 30% of the corresponding tumor type. **^a^**Average tag count count per individaul tunor SAGE library. **^b^**Average induction factor.
